# *In Vitro* AuNPs' Cytotoxicity and Their Effect on Wound Healing

**DOI:** 10.5772/61132

**Published:** 2015-01-01

**Authors:** Veronika Pivodová, Jana Franková, Adéla Galandáková, Jitka Ulrichová

**Affiliations:** 1 The Department of Medical Chemistry and Biochemistry, Faculty of Medicine and Dentistry, Palacky University Olomouc, Czech Republic; 2 The Institute of Molecular and Translational Medicine, Olomouc, Czech Republic

**Keywords:** Gold nanoparticles (AuNPs), Anti-inflammatory activity, Cytotoxicity, Cytokines

## Abstract

Recently, due to their unique properties, gold nanoparticles (AuNPs) have been used in many biological applications. However, little is known about their toxicity when they come into contact with a biological system. Based on the proposal that AuNPs can have a positive effect on wound healing, the present study investigated the influence of negatively-charged-surface AuNPs (average diameter 25–50 nm) on the viability of normal human dermal fibroblasts (NHDF) and normal human epidermal keratinocytes (NHEK). Moreover, we evaluated the effect of AuNPs on the secretion of proteins involved in wound healing, such as interleukin-8 and – 12 (IL-8, IL-12), tumour necrosis factor-alpha (TNF-α), vascular endothelial growth factor (VEGF), basic fibroblast grow factor (bFGF), and granulocyte-macrophage colony-stimulating factor (GM-CSF). The results showed that AuNPs were not toxic to NHDF and NHEK. They showed a decrease in AuNPs' production of pro-inflammatory cytokines IL-6, IL-12 and TNF-α, as well as proteins involved in angiogenesis such as VEGF and bFGF. Thus, we suggest that AuNPs could have anti-inflammatory and anti-angiogenic activity.

## 1. Introduction

Today, nanomaterials are of interest in many scientific fields because they have important applications in a wide variety of areas. They are already used in many commercially available products, such as cosmetics and sunscreens, pharmaceuticals, sports equipment, dressings for wound healing, etc. [1, 2]. Nanomaterials have excellent biological properties, which can make them suitable for medical purposes. In particular, nanomaterials can interact with cellular proteins and organelles. These interactions can be used for the development of new medical and pharmaceutical materials [3].

AuNPs, due to their unique surface, electronic and optical properties, have been often studied [4]. In recent years, AuNPs have served as vehicles for gene delivery, in biosensors, cancer-cell imaging and drug delivery [3, 5]. In addition, it has been reported that AuNPs conjugated by β-cyclodextrin and curcumin could be useful as therapeutic agents for preventing and treating osteoporosis, as they inhibit the formation of tartrate-resistant acid phosphatase (TRAP)-positive multinuclear cells in bone marrow-derived macrophages [6].

However, toxicological studies suggest that AuNPs may cause adverse health effects [7]. Findings have shown that AuNPs may penetrate unusually deep into skin and other organs [8]. It has also been shown that the major pathway of 45 nm AuNPs is mediated by endocytosis and 13 nm AuNPs by phagocytosis [9, 10]. Very small AuNPs (1 nm) can penetrate both into cells and into nuclear membranes [11]. However, the effects of AuNPs on cells are still unclear [8]. It is important to focus on the study of cytotoxicity [12]. Many studies have reported non-toxicity of AuNPs [13, 14], but others have described a toxic effect on cells [7, 15]. It is known that metallic Au is not toxic, but gold chloride or potassium gold cyanide is toxic to organs [16]. Lu et al. (2010) investigated the effect of 34 nm AuNPs in different concentrations on keratinocyte proliferation. It was shown that low concentrations (5 ppm) could stimulate keratinocyte proliferation but that higher concentration (>10 ppm) could be toxic to keratinocytes [17].

It has been shown that AuNP sizes correlate to both the secretion of pro-inflammatory cytokines and antibody production. Spherical (40 nm) AuNPs stimulated proinflammatory cytokines IL-6, IL-12 and TNF- α at a high level. On the other hand, smaller spherical AuNPs (20 nm) did not influence the production of cytokines [18]. It is also well documented that drugs containing AuNPs have anti-inflammatory activities, and, combined with antioxidants, accelerate cutaneous wound healing through anti-inflammatory and antioxidant effects [5, 19].

In this work, we tested the effect of spherical AuNPs (average diameter 25–50 nm) on cell viability and on biomarkers involved in wound healing on normal human epidermal keratinocytes (NHEK) and normal human dermal fibroblasts (NHDF).

## 2. Experiment

### 1.1 Materials

#### 1.1.1 Chemicals

Keratinocyte basal medium-2 and KGM™-2 SingleQuots™ were purchased from East Port Prague s.r.o. (Czech Republic). EpiLife® Medium and Human Keratinocyte Growth Supplement Kits (HKGS) were purchased from Life Technologies s.r.o. (Czech Republic). A human total MMP-1 ELISA kit was purchased from R&D Systems (USA). A development IL-6 ELISA kit and a development IL-8 ELISA kit were purchased from Peprotech (UK). A Bio-Plex Human Cytokines ELISA kit and a Bio-Plex 200 System were purchased from Bio-Rad (USA). Dulbecco's modified Eagle's medium, Ham-F12 Nutrient Mixture, heat-inactivated foetal calf serum, stabilized penicillin-streptomycin solution, amphotericin B, hydrocortisone, adenine, insulin, epidermal growth factor, 3,3‘,5-triiod-L-thyronin, trypsin, ampicillin, amino acids (L-histidine, L-isoleucine, L-methionine, L-tryptophan, L-tyrosine), 3-(4,5-dimethylthiazol-2-yl)-2,5-diphenyltetrazolium bromide (MTT) and all other chemicals were purchased from Sigma-Aldrich (USA).

### 1.2 Methods

#### 1.2.1 Preparation of AuNPs

The sample of AuNPs was obtained from NanoTrade Company and prepared according to the Czech Patent No. 304 160 [20]. Conventional techniques for aqueous synthesis of AuNPs involve reduction of gold chloride (Au (III) Cl3) with trisodium citrate. In this method, the citrate salt initially acts as a reducing agent by forming a layer of citrate ions over the AuNP surface, inducing enough electrostatic repulsion between individual particles to keep them well dispersed in the medium. Briefly, chloroauric acid (HAuCl_4_) was heated to boil. Then, aqueous solution of 1% trisodium citrate was added to the HAuCl_4_ solution under vigorous stirring and the AuNPs were formed by crystallization. The citrate ions serve as reducing agent for AuNP formation and as stabilizer, preventing agglomeration of AuNPs.

#### 1.2.2 Material Characterization

**Figure 1.**
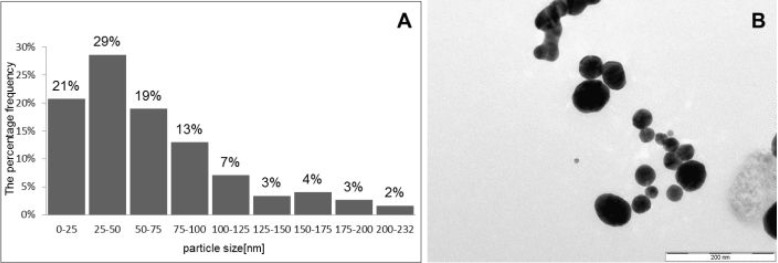
A Particle size distribution; B characterization of AuNPs by transmission electron microscopy (TEM)

Analysis of AuNPs was performed on a JEOL JEM 2011 (JEOL, Tokyo, Japan) at accelerating voltage of 160 kV. AuNPs had an average diameter of 25–50 nm (Fig. 1A). The measurement of Zeta potential was performed using a Brookhaven ZetaPlus Zeta Potential Analyzer (Artisan Technology Group). AuNPs had a negative charge because their Zeta potential was – 23.4 mV. AuNPs stabilized with citrate were characterized by transmission electron microscopy (TEM) (Fig. 1B). Photographs were taken using a Keen View II digital camera using the program iTEM (SIS, Olympus).

#### 1.2.3 Cell Culture

The cells were isolated from the skin specimens, which were removed during abdominoplasty or breast surgery of healthy adult women with their written informed consent and the approval of the Ethics Committee of the Faculty Hospital in Olomouc.

*Normal human dermal fibroblasts (NHDF)*: The samples of skin were cut into pieces (approximately 1×1 cm), placed in Petri dishes (10 cm diameter) and cultured in a mixture of Dulbecco's modified Eagle's medium (DMEM) and Ham's F12 Nutrient Mixture (1:3) supplemented with 10% foetal bovine serum (FBS), penicillin (100 mg/ml), streptomycin (100 U/ml), amphotericin B (0.125 mg/ml), hydrocortisone (0.8 μg/ml), adenine (24 μg/ml), insulin (0.12 U/ml), epidermal growth factor (1 ng/ml), and 3,3′,5-triiod-L-thyronin (0.136 μg/ml). The skin pieces were incubated in humidified atmosphere with 5% CO_2_ at 37°C. The medium was changed every other day until the fibroblasts reached confluence. After two to three weeks, cells were trypsinized and transferred into 75 cm^2^ cultivation flasks. Cells were cultivated in DMEM supplemented with 10% FBS, penicillin (100 mg/ml) and streptomycin (100 U/ml) at $37^{\underline{o}}C$ in a 5% CO2 atmosphere. Cells were used between splitting 2 and 3.

*Normal human epidermal keratinocytes (NHEK)*: The samples of skin were cut into pieces (approximately 2×3 cm), placed into cold 0.5% trypsin solution and incubated overnight at $4^{\underline{o}}C$. After the incubation period, the epidermis and dermis were separated using thin forceps and the epidermal tissue was placed in the keratinocyte basal medium-2 (KBM-2) supplemented with KGM™-2 SingleQuots™ and 2% FBS. The epidermal cells were then dissociated mechanically by stirring. The cell suspension was filtered through a cell strainer and centrifuged (5 min, 1000 rpm, $4^{\underline{o}}C$). The pellet was resuspended in KBM-2 supplemented with KGM™-2 and FBS and transferred into 75 cm^2^ cultivation flasks; cells were then grown in humidified atmosphere for three days. After the incubation period the KBM-2 supplemented with KGM™-2 and FBS was altered by the EpiLife® growth medium (EPIM) supplemented with a Human Keratinocyte Growth Supplement Kit (HKGS), penicillin (100 mg/ ml), streptomycin (100 mg/l) and ampicillin (250 μg/ml) at $37^{\underline{o}}C$ in a 5% CO2 atmosphere. The medium was changed every other day until the keratinocytes reached 50–60% confluence. Cells were used at splitting 3 [21].

#### 1.2.4 Cell Viability

Cells were seeded on 96-well culture plates (NHDF at concentration 1×10^5^ cells/cm^2^, NHEK at concentration 8×10^3^ cells/cm^2^). After 24 hrs, AuNPs diluted in serum-free DMEM (NHDF; concentration range 0.01–25 ppm) and serum-free EPIM (NHEK; concentration range 0.013–33 ppm) were applied to cells and incubated for 24 hrs. Control cells were incubated in serum-free medium without AuNPs. MTT assay was used to evaluate cell survival. This assay is based on the ability of viable cells to convert soluble methyl tetrazolium salts to insoluble formazan. Formazan is quantitated by spectrophotometry after being dissolved in dimethyl sulphoxide and ammonia. The absorbance was measured at 540 nm.

#### 1.2.5 Wound Scratch Assay

The cells were seeded on six-well culture plates (NHDF at concentration 5×10^5^ cells/cm^2^, NHEK at concentration 3×10^4^ cells/cm^2^) and cultivated to form a confluent mono-layer. Then, the cells were scratched with a sterile 10 ml plastic pipette. The cells were washed with PBS and AuNPs were added in non-toxic concentrations (0.25 ppm; 2.5 ppm; 25 ppm) diluted in corresponding serum-free medium. The control cells were cultured in corresponding serum-free medium without AuNPs.

After 24 and 48 hrs the cell supernatant was collected for determination of interleukin-12 (IL-12), tumour necrosis factor-alpha (TNF-α), basic fibroblast grow factor (bFGF), vascular endothelial growth factor (VEGF) and granulocyte-macrophage colony-stimulating factor (GM-CSF) by Bio-Plex analysis; matrix metalloproteinase-1 (MMP-1), interleukin-6 (IL-6) and interleukin (IL-8) were determined by ELISA kits. All markers were determined according to manufacturer protocol.

#### 1.2.6 Statistical Analysis

The results were expressed as mean ± SD of five independent experiments. The differences in mean values were analysed by Student's t-test. *P < 0.05; **P < 0.01 or ***P < 0.001 were considered statistically significant.

## 3. Results and Discussion

The interaction of nanoparticles (NPs) with biological systems including living cells has become one of the most crucial areas of research in materials science and biology.

In our study the effect of spherical AuNPs (average diameters of 25–50 nm) on cell viability and on biomarkers involved in wound healing in NHDF and NHEK was tested. Accurate size and shape of AuNPs were determined by transmission electron microscopy (TEM) (Fig. 1B). The TEM analysis revealed that AuNPs have spherical morphology (Fig. 1). The size distribution analysis showed 29% of the AuNPs occurring between 25–50 nm in size (Fig. 1A).

**Figure 2.**
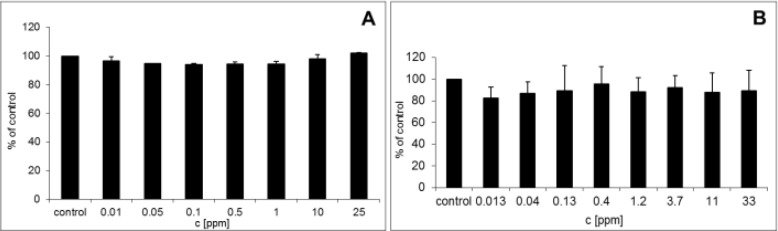
A Cytotoxicity of AuNPs on NHDF; B cytotoxicity of AuNPs on NHEK

It was shown that AuNPs have many side effects due to the interaction with cell membrane, mitochondria, or nucleus. One recent study has shown toxicity of AuNPs to be related to dose, surface charge and area [22]. It has also been reported that the surface charge is one of the major factors influencing AuNPs' toxicity [23]. Several studies have compared the toxicity of AuNPs (2 nm) with positively and negatively charged surface. It has been found that negatively charged AuNPs had IC50 >7.37 μmol/l for monkey fibroblast cell lines (Cos-1) and IC50 for red blood cells was 72 μmol/L while positively charged ones had IC50 1 μmol/l for both types of cell under the same condition. The higher toxicity of positively charged AuNPs was explained by their ability to interact with the negatively charged cellular membrane [15]. On the other hand it was demonstrated that AuNPs (1.5 nm) charged both positively and negatively were toxic on HaCaT cell lines at the concentration of 10 μg/ml [24]. In our study toxicity of negatively charged AuNPs (-23.4 mV) was evaluated by cell viability MTT assay after 24 hrs. It was shown that AuNPs do not have a significant cytotoxic effect on NHDF (0.01–25 ppm) (Fig. 2A) and NHEK (0.013–33 ppm) (Fig. 2B) at any concentrations. However, NHEK was more sensitive to AuNPs because they showed lower viability compared to NHDF.

Others factors, such as ionic strength, size, shape and stabilizer can also affect the toxicity of AuNPs. It has been found that spherical AuNPs (21 nm) stabilized by citrate does not have a toxic effect on human breast-cancer cell lines (MCF-7) or human prostate-cancer cell lines (PC-3). It has also been reported that spherical AuNPs (10–50 nm) stabilized by citrate were not toxic to human leukemic cells (K562) and NHDF. These results are in line with our findings whereby spherical AuNPs (average diameter of 25–50 nm) stabilized by sodium citrate were not significantly toxic to NHDF or NHEK [25, 23].

Some studies have demonstrated that AuNPs induce the release of a number of pro-inflammatory markers in bonemarrow dendritic cells and in macrophages. *In vivo* studies have shown that AuNPs exhibited anti-inflammatory properties via decreasing of the pro-inflammatory cytokines such as IL-6 and TNF-α due to the inhibition of nuclear factor kappa-B (NF-kB) activation [26, 27].

**Figure 3.**
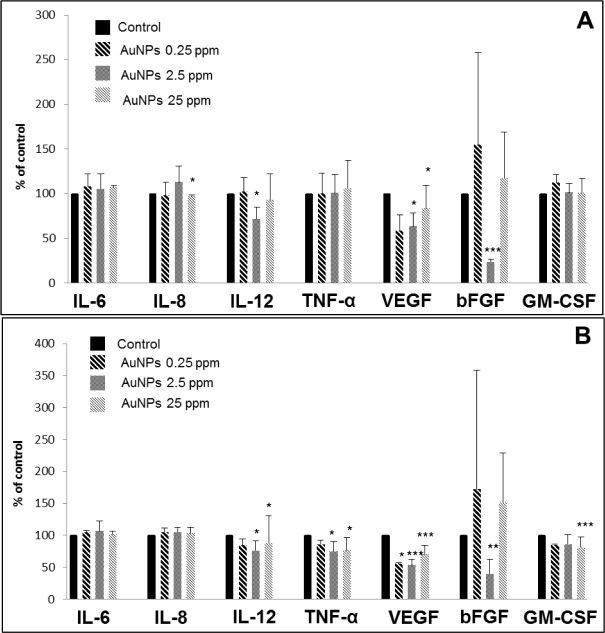
Effects of AuNPs on wound-healing markers in NHDF after 24 hrs (A) and 48 hrs (B). Control was scratch cells incubated in serum-free medium without AuNPs; an asterix (*) indicates statistically significant difference from the control (*p<0.05; **p<0.01; ***p<0.001).

**Figure 4.**
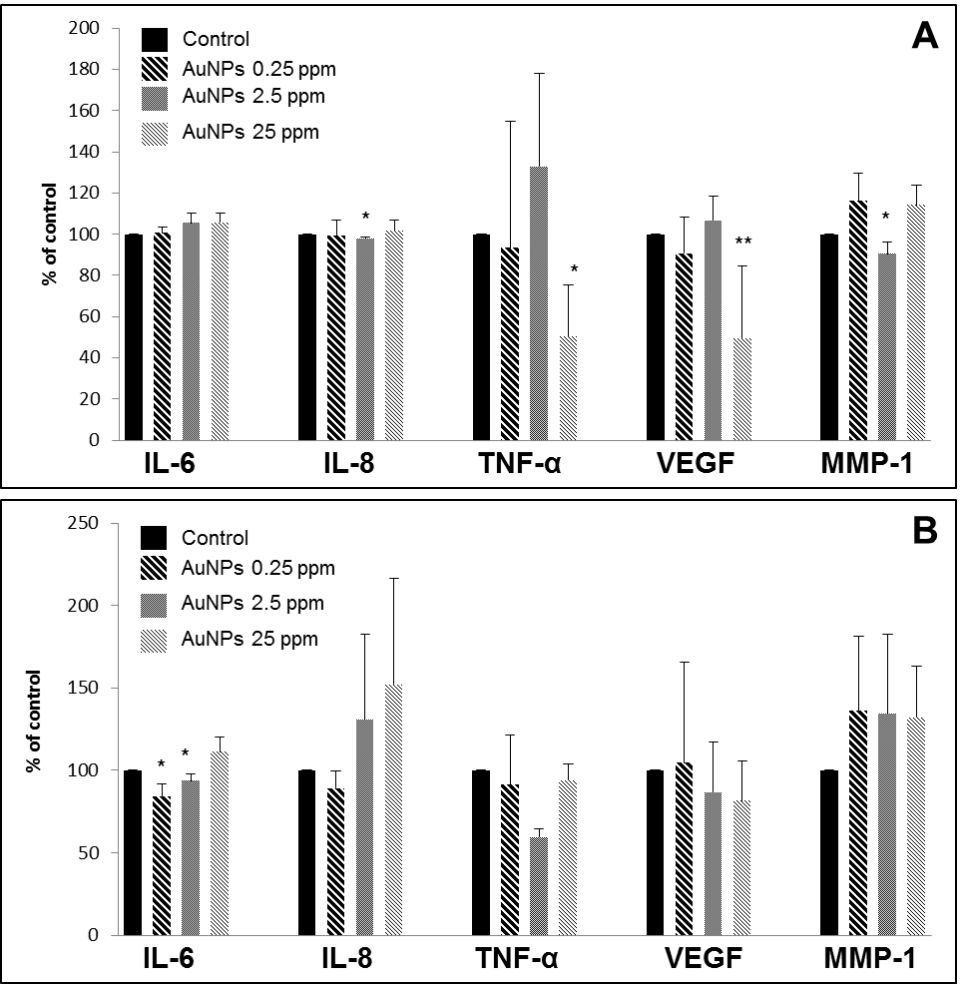
Effects of AuNPs on wound-healing markers in NHEK after 24 hrs (A) and 48 hrs (B). Control was scratch cells incubated in serum-free medium without AuNPs; an asterix (*) indicates statistically significant difference from the control (*p<0.05; **p<0.01).

AuNPs may also have characteristic properties according to their size. It has also been shown that AuNPs (40 nm) stimulated pro-inflammatory cytokines IL-6, IL-12 and TNF-α, but smaller AuNPs (20 nm) did not induce any intense changes in the levels of pro-inflammatory markers [18]. Our results showed that AuNPs (25–50 nm) were more active in NHDF (Fig. 3) than in NHEK (Fig. 4). It was found that AuNPs at the concentration of 2.5 ppm decreased the production of IL-12, VEGF and bFGF after 24 hrs (Fig. 3A) and IL-12, VEGF, bFGF, and TNF-α after 48 hrs (Fig. 3B) in NHDF. Moreover, higher concentrations of AuNPs (25 ppm) decreased level of IL-8 and VEGF after 24 hrs (Fig. 3A) and IL-12, VEGF, GM-CSF and TNF-α after 48 hrs (Fig. 3B) in NHDF. Meanwhile, in NHEK AuNPs at concentrations of 2.5 and 25 ppm were effective only after 24 hrs. It was found that AuNPs at 2.5 ppm decreased the production of IL-6 and MMP-1 and the higher concentration of 25 ppm decreased levels of VEGF and TNF-α (Fig. 4A, B). However, in our work, the size of AuNPs is not uniform. Therefore, we cannot compare our results in terms of size of AuNPs.

Besides anti-inflammatory activity, AuNPs were found to have anti-angiogenic effects. It has been shown that AuNPs interact with the sulphur/amines present in the heparin-binding domain of VEGF_165_ [28]. It has also been indicated that AuNPs inhibit VEGF_121_ by changing its conformation [29]. These results are in agreement with our study, where we determined that AuNPs decrease the level of VEGF in both cell cultures, NHDF and NHEK. Moreover, it is known that bFGF is an angiogenic agent and has affinity for heparin like VEGF [30]. We found that AuNPs also decreased the level of bFGF in NHDF. This effect of AuNPs may be beneficial for the treatment of cancer due to the inhibition of angiogenesis.

## 4. Conclusion

Our study showed that AuNPs (average diameter 25–50 nm) with negatively charged surface (-23.4 mV) were not toxic to NHDF and NHEK. In addition, they exerted anti-inflammatory activity via inhibition of IL-6, IL-12 and TNF-α level, and anti-angiogenic activity decreasing the expression of VEGF and bFGF. These properties are of clinical significance and contribute to the application of AuNPs in biomedicine. The anti-angiogenic activity can be exploited for cancer therapy [10]. In addition, cutaneous wounds, diabetic wound tissue, and rheumatoid arthritis can be treated with AuNPs due to their anti-inflammatory properties [10, 31, 32].
